# Training Issue about Digital Tools : from Users & Carers to Professionals

**DOI:** 10.1192/j.eurpsy.2022.108

**Published:** 2022-09-01

**Authors:** M. Dulière

**Affiliations:** Haute Ecole de la Province de Namur, Département Des Sciences De La Santé Publique Et De La Motricité, Namur, Belgium

**Keywords:** caregivers, training, future healthcare professional, e-health

## Abstract

The Interreg IT4Anxiety Project gives birth to training modules in digital health. The European Interreg IT4Anxiety project, led by the CNP St-Martin, is launching a new range of training modules in digital health awareness at the Haute Ecole de la Province de Namur, the University of Applied Sciences (HEPN). Electronic medical records, teleconsultation, mobile applications, virtual reality… Digital tools are already part of the daily life of workers in the health sector. However, until now, no curriculum has included appropriate training to enable caregivers to acquire the fundamental knowledge needed to use digital tools effectively in their professional practice. Thanks to the expertise and support of its various partners in the IT4Anxiety project, the Haute Ecole de la Province de Namur will provide digital health awareness training starting in 2022. These training modules will be provided as part of the in-situ training of caregivers and are already being tested in the academic training of future nurses. In addition to discovering the potential of new technologies in the health field, health professionals and future professionals will learn to identify the appropriate use of these tools, to deploy them and to use them in their profession, in a respectful and caring relationship with a critical eye. The structure of the courses, the assessment and the feedback from the HEPN will form the basis for an international deployment of this training by the German, French, Dutch and English partners of the IT4Anxiety project.

**Disclosure:**

No significant relationships.

